# A pattern-triggered immunity-related phenolic, acetosyringone, boosts rapid inhibition of a diverse set of plant pathogenic bacteria

**DOI:** 10.1186/s12870-021-02928-4

**Published:** 2021-03-25

**Authors:** Ágnes Szatmári, Ágnes M. Móricz, Ildikó Schwarczinger, Judit Kolozsváriné Nagy, Ágnes Alberti, Miklós Pogány, Zoltán Bozsó

**Affiliations:** 1 Plant Protection Institute, ELKH Centre for Agricultural Research, Herman Ottó St. 15, Budapest, 1022 Hungary; 2 Present address: Chemical Biology Research Group, Institute of Organic Chemistry, ELKH Research Centre for Natural Sciences, Magyar tudósok körútja 2, Budapest, 1117 Hungary; 3 Department of Pharmacognosy, Faculty of Pharmacy, Semmelweis University, Üllői St. 26, Budapest, 1085 Hungary

**Keywords:** Pattern-triggered immunity, *Pseudomonas syringae*, Acetosyringone, Elicitor, Oxidative burst, Antibacterial

## Abstract

**Background:**

Acetosyringone (3,5-dimethoxy-4-hydroxyacetophenone, AS) is a syringyl-type phenolic compound rarely found in plants in free form. It has been shown earlier to inhibit the growth of *Pseudomonas* bacteria in the presence of hydrogen peroxide and peroxidase (AS mix).

**Results:**

We detected elevated levels of free AS in *Nicotiana tabacum* and *N. benthamiana* plants after inducing pattern-triggered immunity (PTI) by injecting bacterial elicitor flg22, or pathogenicity-mutant *Pseudomonas syringae* pv. *syringae* 61 *hrcC*- bacteria; but not after inoculations with compatible or incompatible pathogens at the time of PTI onset. In this study, we demonstrate that the antibacterial effect of the AS mix is general, as growth of several Gram-negative and -positive phytopathogenic bacteria was characteristically inhibited. The inhibition of bacterial metabolism by the AS mix was rapid, shown by the immediate drop of luminescence intensity of *P. syringae* pv. *tomato* DC3000 lx strain after addition of AS mix. The mechanism of the bacteriostatic effect was investigated using fluorescent reporter dye assays. SYTOX Green experiments supported others’ previous findings that the AS mix does not result in membrane permeabilization. Moreover, we observed that the mode of action could be depolarization of the bacterial cell membrane, as shown by assays carried out with the voltage sensitive dye DIBAC_4_(3).

**Conclusions:**

Level of free acetosyringone is elevated during plant PTI responses in tobacco leaves (*N. tabacum* and *N. benthamiana*). When combined with hydrogen peroxide and peroxidase (AS mix), components of the mix act synergistically to inhibit bacterial metabolism and proliferation rapidly in a wide range of plant pathogens. This effect is related to depolarization rather than to permeabilization of the bacterial cell membrane. Similar AS mixture to the in vivo model might form locally at sites of invading bacterial attachment to the plant cells and the presence of acetosyringone might have an important role in the inhibition of bacterial proliferation during PTI.

**Supplementary Information:**

The online version contains supplementary material available at 10.1186/s12870-021-02928-4.

## Background

Similarly to the innate immune response of mammals, plants also have an inducible resistance system called pattern-triggered immunity (PTI). This form of plant resistance recruits pattern recognition receptors (PRRs) that have a structure like Toll-like receptors (TLRs) of mammals. PTI, similarly to innate immune responses, can be triggered by microbe associated molecular patterns (MAMPs). These include a conserved peptide from bacterial flagellin (flg22), elongation factor Tu (EF-Tu), peptidoglycan (PGN), lipopolysaccharides (LPSs), activator of XA21-mediated immunity in rice (Ax21) (reviewed in: [1, 2]). PTI can also be induced *in planta* by non-pathogens: saprophytes, commensals (e.g. *Pseudomonas fluorescens*) or pathogenicity mutants (e.g. *Pseudomonas syringae* pv. *syringae* 61 *hrcC*-) [3–5]. PTI is effective against a broad range of microbes and is able to suppress opportunistic pathogens. Its early signalling events include rapid calcium influx, activation of mitogen activated protein kinase (MAPK) phosphorylation cascades, which then lead to production of reactive oxygen species (ROS), callose deposition, and defence gene expression, presumably mediating inhibition of microbial growth [6]. Disease-inducing, virulent *P. syringae* pv. *tabaci* in tobacco plant is able to block the MAMP-induced expression level alterations of specific genes coding for signal transduction and transcription regulation-related proteins [7].

During our previous work, we identified over 400 expressed sequence tags (ESTs) representing 176 individual genes that are activated during PTI in *Nicotiana tabacum*. Phenylpropanoid synthesis-related genes were highly represented and covered a major part of the currently known network of the phenylpropanoid pathway [8]. Our hypothesis was that elements of the phenylpropanoid pathway might play an important role in the inhibitory effects of PTI on bacteria, either by leading to cell wall fortification or by some phenolic compounds having direct antimicrobial effect, or else, by acting as a signalling agent, affecting virulence factors of bacteria. Lignification and induction of lignin monomers’ and related compounds’ synthesis has long been associated with plant defences [9, 10], and lately with flg22-induced immunity in *Arabidopsis* [11]. Recently, lignin deposition has been shown to spatially restrict bacterial pathogens and to limit their motility also in *Arabidopsis* [12].

Baker and colleagues [13] have detected accumulation of acetosyringone (AS) and some other phenolics in *N. tabacum* cell cultures, when treated with suspensions of *P. syringae* pv. *syringae* strain B7 (non-HR inducing). Later they have also shown that *P. fluorescens,* a non-pathogenic species causes elevation of AS in the apoplast fluid of *N. tabacum* [14]. In this study we describe detection and identification of AS in *N. benthamiana* and *N. tabacum* leaves using high-performance liquid chromatography-diode array detection-mass spectrometry (HPLC-DAD-MS), moreover, we show the MAMP (i.e. flg22) associated accumulation of this phenolic compound, and suggest that it is a marker of PTI in tobacco plants.

We sought for compound(s) that are accumulated during PTI in both *Nicotiana* model species, because while *N. tabacum* is a classical model plant to study bacterial interactions in the *Solanaceae* family, parallel data from *N. benthamiana* might later prove to be useful as well, as transient silencing and overexpression studies are easier to carry out in the latter species. We investigated if appearance of AS is specific for PTI in *N. benthamiana* and whether it correlates temporally with the onset of PTI, using HPLC-DAD-MS. Based on reports of AS as an enhancer of laccase activity [15] and having antibacterial activity when combined with horseradish peroxidase and hydrogen peroxide [16], we tested a wide range of plant pathogenic bacteria to detect antibacterial activity. We also used luminescent *P. syringae* pv. *tomato* DC3000 bacteria to monitor effects on bacterial metabolic activity. Finally, to gain information about the mode of action of AS we used fluorescent dyes to measure membrane permeabilization and membrane depolarization of affected bacteria.

## Results

### Identification of acetosyringone, a metabolite correlated with PTI onset

Our first aim was to detect and identify phenolic metabolite that are specifically more (or less) abundant in *N. benthamiana* leaves in association with the onset of PTI. *N. benthamiana* leaves were infiltrated with *Pseudomonas syringae* pv*. syringae hrcC*- (*P. s. syringae hrcC*-) suspension or flg22 peptide to induce PTI, and water as a control. Both PTI inducers were used at concentrations that caused reliable HR-inhibition at 6 h post inoculation (flg22 at 50 μM, *P. s. syringae hrcC*- at 10^9^ CFU/ml), based on works of Nguyen et al. and Chakravarthy et al. [17, 18]. Inhibition of HR, sometimes referred to as an “HR test” is an indicator of the onset of effective PTI and correlates well with the onset of local resistance that is able to halt bacterial proliferation after a challenge inoculation [3, 8, 17]. Leaf samples were taken 6 h later and extracts were analyzed by HPLC-DAD-MS to find PTI-related phenolic compounds. Extraction from *N. benthamiana* leaves was performed with 90% aqueous methanol according to recommendations in literature [19].

**Fig. 1:**
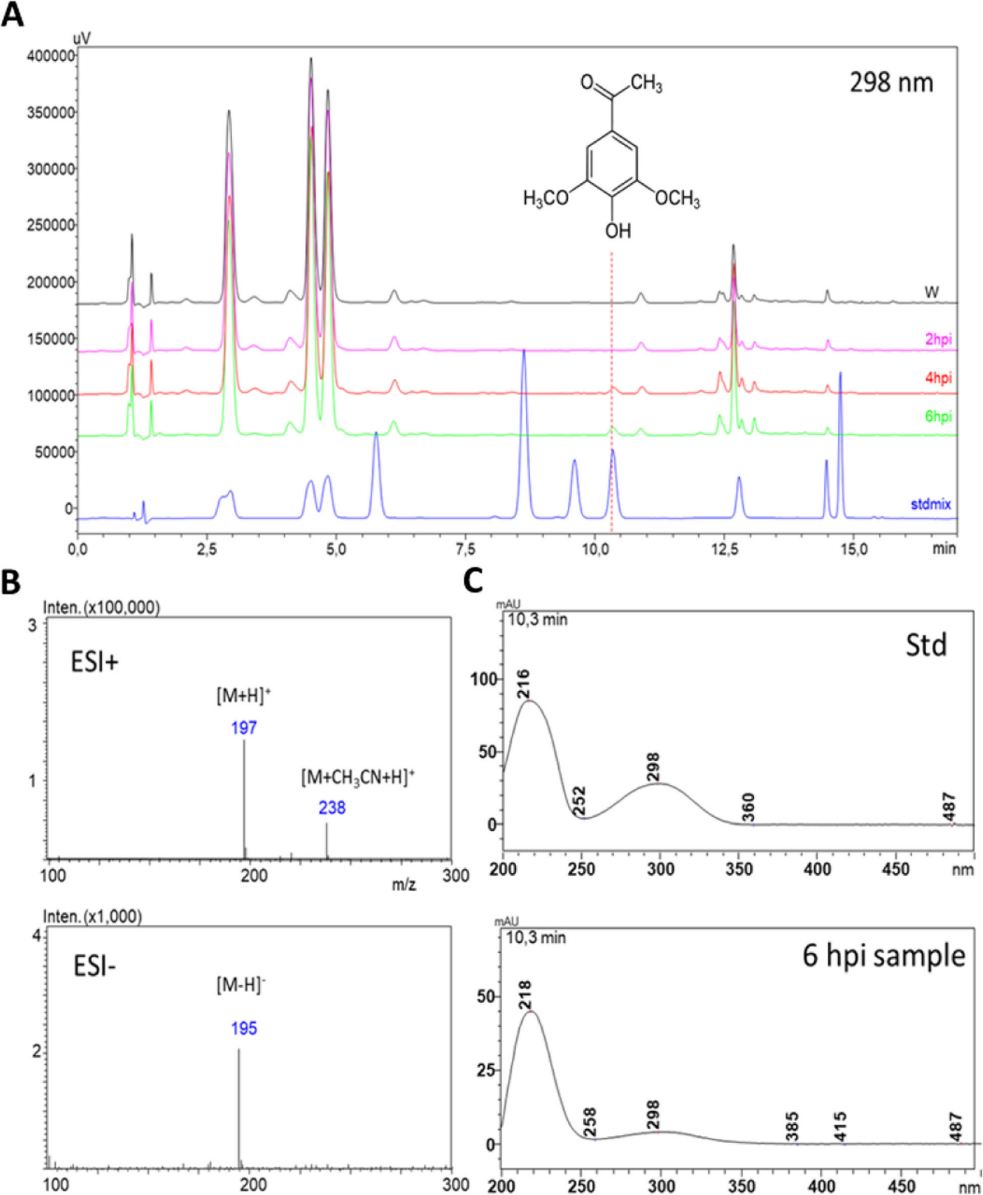
Identification of PTI-related phenolic compound acetosyringone by HPLC-DAD-MS using analytical standard in *Nicotiana benthamiana*. **a** HPLC-DAD analysis of phenolic compounds from PTI-induced and water-treated control *N. benthamiana* leaves detected at 298 nm. PTI was induced by *P. syringae* pv*. syringae hrcC*- bacteria, water was injected as control. Samples were taken at 2, 4, 6 hpi. **b** MS spectra of AS had the expected *m/z* values in both positive and negative modes, identical to that of the standard. **c**) Identity of acetosyringone was supported by the similar UV spectra of the analytical standard and the unknown peak. Abbreviations: AS: acetosyringone, ESI+, ESI-: electrospray ionization positive and negative modes, Std: standard, W: water

We detected several putative phenolic compounds (with characteristic UV spectra) in PTI-induced *N. benthamiana* leaves (data not shown). Works of others [13, 20] carried out on *N. tabacum* helped identification of some of these compounds. In this manuscript, we focus on one specific compound (*m/z* 197 [M + H]^+^) that occurred selectively in PTI-induced (6 h post inoculation, *hpi*) *N. benthamiana* leaves (Fig. 1a), and was identified as acetosyringone (3,5-dimethoxy-4-hydroxyacetophenon), by comparison of its MS (Fig. 1b) and UV (Fig. 1c) spectra and retention time (Fig. 1a) to those of an analytical standard. Spiking the standard compound into the plant extracts resulted in clear elevation of the putative AS peak.

**Fig. 2:**
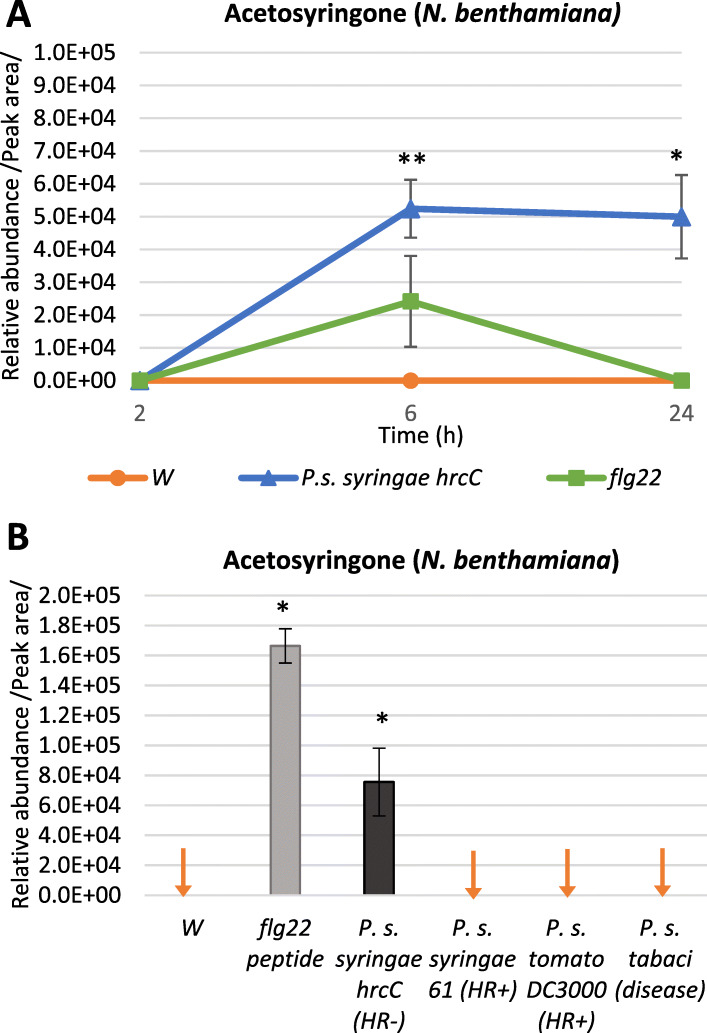
Relative accumulation of acetosyringone in *N. benthamiana* leaves. **a** Response to treatments with *P. syringae* pv. *syringae hrcC*- bacteria at 2, 6 and 24 hpi. **b** Relative accumulation of AS in *N. benthamiana* leaves in response to treatments with different bacteria at 6 hpi. Error bars indicate standard deviations. Asterisks indicate significant difference from corresponding water-treated controls according to student’s T-test (*p < 0.1; **p < 0.05). Red downward arrows indicate zero values. W: water-treated control; P. s.: *Pseudomonas syringae* pathovars; flg: flg22 peptide elicitor

We found AS especially interesting, because besides *P. s. syringae hrcC*- treatment, it was also strongly induced in flg22 peptide-treated *N. benthamiana* leaves, but was not detectable in water treated controls at 6 hpi (Fig. 2a). The abundance of AS correlated closely with PTI in the leaves of both *N. benthamiana* (Fig. 2a), and tobacco (*N. tabacum,* Additional file 1). AS itself was first isolated from *N. tabacum* leaf exudates and root culture medium, where it was established to be exudate specific, not passively leaking out from damaged plant cells [21]. Baker et al. have reported accumulation of AS in tobacco cell culture media [13] and in the apoplastic fluid [14] after treatment with different bacteria.

### Changes in phenolic metabolite levels

To see if elevated level of AS occurs in different plant-bacterium interactions, we analyzed 6 hpi samples from *N. benthamiana* leaves injected with suspensions of a compatible bacterium: *Pseudomonas syringae* pv. *tabaci*, two incompatible bacteria: *P. syringae* pv*. tomato* DC3000 and *P. syringae* pv*. syringae* 61, PTI inducing bacterium: *P. syringae* pv*. syringae hrcC*- (10^9^ CFU/ml each), and a PTI-inducing MAMP: flg22 peptide. Water-treated controls were also analyzed. Interestingly, AS displayed a unique pattern: it was only detected in flg22 and *P. syringae* pv*. syringae hrcC-* treated samples – the two PTI- induced samples; but it was absent (below detection limit) in the rest of the samples at the used concentrations (Fig. 2b). Time course experiments supported the gradual accumulation of AS during the course of the build-up of effective PTI both in *N. benthamiana* (Fig. 2a) and *N. tabacum* (Additional file 1). It should be noted that there appears a co-timing with inhibition of HR and of proliferation of compatible bacteria upon challenge inoculation; which usually becomes effective also at 5–6 h after induction of PTI [22].

### Antimicrobial effect of acetosyringone in combination with hydrogen peroxide and peroxidase against plant pathogenic bacteria

Several sources reported that AS or related acetophenones have antifungal or antibacterial effects. Lorimer and Perry [23] reported isolation of two major antifungal active components from *Plagiochila fasciculata* (New Zealand liverwort). These were identified as 2-hydroxy-4,6-dimethoxyacetophenone and 2-hydroxy-3,4,6-trimethoxyacetophenone. The relatively high activity of the crude extract was due to the high level of these compounds in the plant (6–7 mg/g dried plant for both). In vitro oxidation of AS with hydrogen peroxide by a peroxidase can create a prolonged oxidative environment similar to the oxidative burst in tobacco suspension cells inoculated with incompatible bacteria according to Mock et al. [16].

**Fig. 3:**
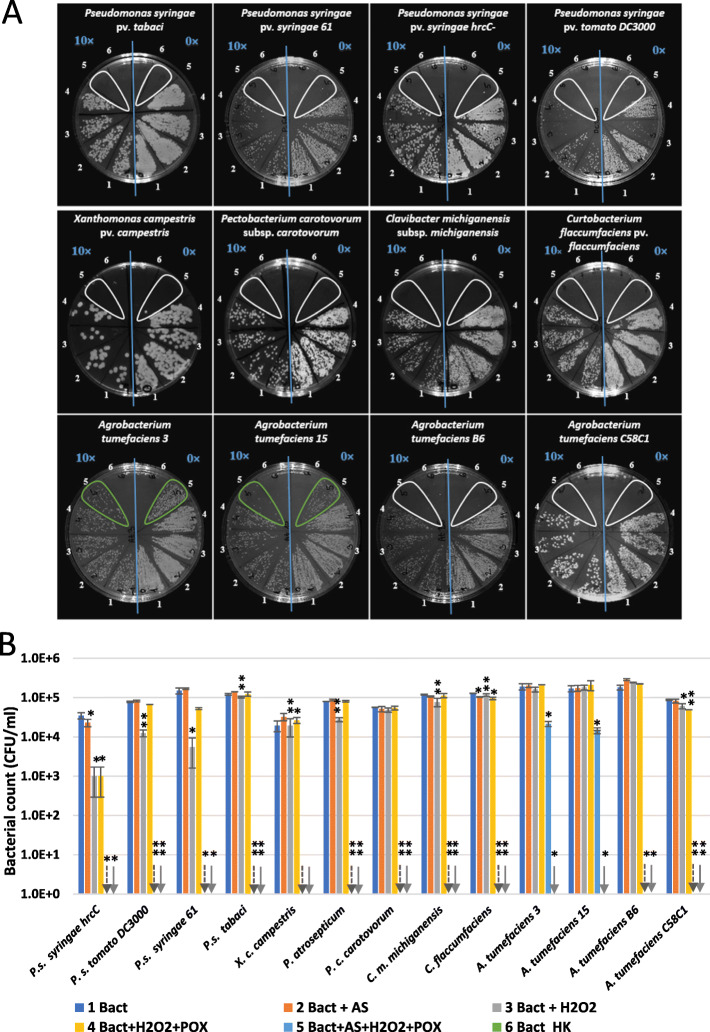
Antimicrobial effect of oxidized form of acetosyringone on different plant pathogenic bacteria. Bacteria (10^5^ CFU/ml) were added to reaction mixtures containing 50 μM AS, 50 μM H_2_O_2_, and 0.72 U/ml horseradish peroxidase, and various control mixtures from which one or two components were omitted. Serial dilutions were plated following 3 h of co-incubation for CFU determination. **a** Typical representative examples of serial dilutions plated on Kings’s B agar plates. Right half of each plate: no dilution (0×), left side of each plate: 10× dilution (indicated with blue lettering). Numbering of treatment combinations applied to the bacterial suspensions: 1. non-treated control; 2. AS; 3. H_2_O_2_; 4. H_2_O_2_ + POX; 5. AS + H_2_O_2_ + POX; 6. HK. **b** Diagram showing quantification of the results. Error bars indicate standard deviations. Asterisks indicate significant difference from corresponding water-treated controls according to student’s T-test (*p < 0.1; **p < 0.05). Downward arrows indicate zero values. Abbreviations: Bact: bacterium, AS: acetosyringone, HK: heat-killed, POX: horseradish peroxidase

Based on the latter, we tested if in vitro oxidation of AS results in antimicrobial effects against bacterial strains corresponding to different plant (tobacco)-bacterium interactions (Fig. 3a-b). *P. s.* pv. *tomato* DC3000 and *P. s.* pv. *syringae* 61, and the PTI-inducing bacterium *P. s.* pv. *syringae hrcC*- and other plant pathogenic bacteria were tested, including wild type tumorigenic *Agrobacterium tumefaciens* strains (from cherry, sour cherry and dahlia) [24] and a disarmed laboratory strain (*A. tumefaciens* C58C1) [25]. *V*irulence genes of *Agrobacteria* are activated by AS, and they are known to be poor inducers of PTI marker genes (Szatmári et al. 2006). We combined 50 μM AS, 50 μM H_2_O_2_ and 0.72 U/ml peroxidase in phosphate buffer, containing 10^5^ CFU/ml suspensions of different bacteria. Serial dilutions were plated after 3 h of co-incubation. No growth of the *Pseudomonas*, *Xanthomonas*, *Pectobacterium*, *Clavibacter* or *Curtobacterium* strains was detected after 3 h of incubation in the complete reaction mixture. On the contrary, *Agrobacterium* growth was retained in the case of two of the examined strains. When omitting different components of the mixture, some *Pseudomonas* strains were still inhibited to various extent. For example CFU counts of *P. s.* pv. *syringae hrcC*- were lowered by one order of magnitude when treated with H_2_O_2_ and peroxidase. However, when the mixture was completed with AS, no growth was detected at all. AS alone was not effective against any of the strains, so the combination of acetosyringone with peroxidase activity is essential. Activation of tobacco peroxidase activity *in planta* at the time interval of AS production during PTI has been shown earlier [3].

When identical concentrations of AS, H_2_O_2_ and peroxidase were combined with higher concentrations of bacteria, the mixture was not effective in CFU reduction. At 5 × 10^7^ CFU/ml *Pseudomonas syringae* pv. *tabaci* and *Agrobacterium tumefaciens* C58C1 were apparently unaffected, and at 5 × 10^6^ CFU/ml they only showed a 5–6-fold reduction in CFU counts (Additional file 2). There seems to be a strong factor depending on bacterial concentration that seems to counter the effect of oxidized AS.

We were curious about the changes occurring in the AS mix after combining the ingredients. We found that the amount of AS is diminishing over time only in the full AS mix, but not in the partial mixes containing AS+POX or AS+H_2_O_2_ (Additional file 3A). New compounds appeared only in the latter throughout the 180 min period (Additional file 3B).

The LC–mass spectrometry analysis of the full AS mix reaction yielded m/z peaks corresponding to AS itself (m/z = 197, [M + H]+; (m/z = 195, [M-H]-); putative compounds syringaldehyde (m/z = 183, [M + H]+; (m/z = 181, [M-H]-) and 2,6-DMQB (2,6-dimethoxy-p-benzoquinone, m/z = 169, [M + H]+) and several other peaks corresponding to unknown compounds (Additional file 3B). Putative identification of the newly formed compounds was based on data from literature [26, 27].

The catalytic cycle of oxidases like peroxidases and laccases in general involves an intermediate phenoxy radical after phenolic substrates release a proton and an electron when they are oxidized in the enzymatic step to produce radicals. The phenoxy radicals can be involved in the radical recombination, cross-coupling and self-coupling resulting in disproportionation, dimerization, polymerization, or oxidation of other substrates [26, 28]. The resulting compounds are expected to be the products of such reactions.

**Fig. 4:**
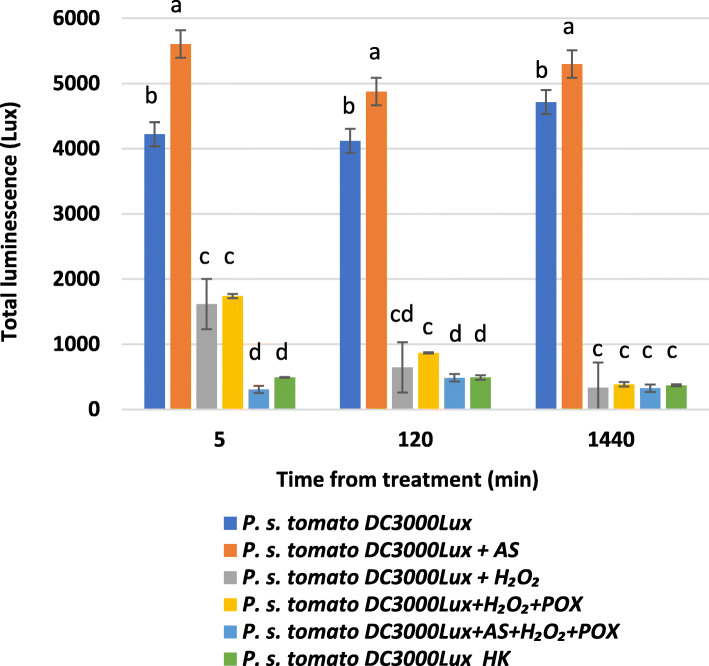
Bioluminescence changes of indicator bacteria after treatment with oxidized AS. 10^5^ CFU/ml of *P. syringae* pv. *tomato* DC3000 lux, a luminescent *P. syringae* strain was added to reaction mixtures containing 50 μM AS, 50 μM H_2_O_2_, and 0.72 U/ml POX, and various control mixtures from which one or two components were omitted. Bioluminescence was measured at indicated time points. Different letters above the bars denote different levels of luminescence assigned by Tukey’s Test (*P* < 0.05). Error bars indicate standard deviations. AS: acetosyringone, HK: heat-killed, *P.s. tomato* DC3000 lux: *Pseudomonas syringae* pv. *tomato* DC3000 luminescent strain, POX: horseradish peroxidase

We used a luminescent *P. syringae* pv. *tomato* DC3000 strain that is tagged chromosomally with the *luxCDABE* gene cluster [29] to monitor the changes in bacterial cell viability caused by oxidized AS. Viable *P. s.* pv. *tomato* DC3000 lux bacteria actively emit a constant level of luminescence. We planned to monitor the time dependent decrease of luminescence, when combined with oxidized AS. We found that the luminescence level dropped virtually immediately to the background level of heat killed bacteria already at the first measurement (5 min) after administration of the AS + H_2_O_2_ + POX cocktail (Fig. 4). This response was significantly faster than the effect caused by H_2_O_2_ alone or H_2_O_2_ combined with peroxidase. AS alone did not reduce bioluminescence. In fact, it seemed to significantly increase bioluminescence compared to untreated bacteria, a phenomenon that needs further investigations to find an explanation. Altogether, these results implied that metabolic activity of the bacteria was halted immediately after addition of the AS + H_2_O_2_ + POX cocktail. Therefore, not only is the mixture more effective in inhibiting bacterial proliferation than H_2_O_2_ + peroxidase alone, but acts much quicker on the bacteria, at least as far as metabolic activity is concerned.

### Testing viability and membrane depolarization of acetosyringone mix treated bacterial cells by enhanced fluorescence of selected dyes

**Fig. 5:**
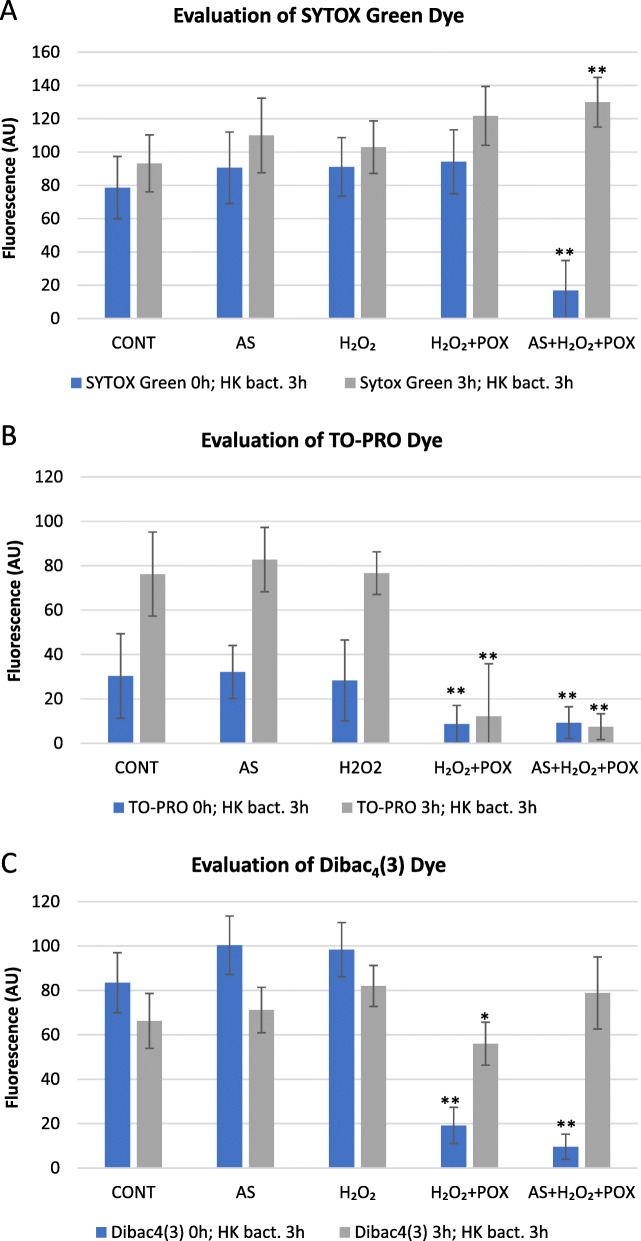
Evaluation of applicability of different fluorescent dyes for the detection of membrane permeability and membrane depolarisation in the AS mix system. Different fluorescent dyes were tested for usability to measure membrane permeability: SYTOX Green (**a**), TO-PRO (**b**); and membrane depolarisation: DIBAC_4_(3) (**c**). Fluorescence of indicator dyes in reaction mixtures containing 50 μM AS, 50 μM H_2_O_2_, and 0.72 U/ml POX, and various control mixtures from which one or two components were omitted was measured. Fluorescent dyes were added either immediately (0 h) or 3 h after preparation of the mixtures to see if bleaching occurs. HK *P. syringae* pv. *tabaci* suspension was also added 3 h after mixture preparation to 5 × 10^6^ CFU/ml final density. Fluorescence was measured 4 h after preparation of the mixtures. Error bars indicate standard deviations. Asterisks indicate significant difference from corresponding controls according to student’s T-test (*p < 0.1; **p < 0.05). Abbreviations: CONT: untreated bacteria, AS: acetosyringone, HK: heat-killed, POX: horseradish peroxidase, AU: arbitrary unit

We tested several fluorescent dyes to be able to detect viability of bacteria upon treatment with AS mixture (Fig. 5a-c). The SYTOX Green and TO-PRO-3 iodide assays are generally used to assess bacterial cell membrane integrity, while DIBAC_4_(3) is known to be able to report slower speed membrane depolarization. With DIBAC_4_(3) and TO-PRO-3 iodide we found that the H_2_O_2_ + POX and AS + H_2_O_2_ + POX mixtures caused bleaching of the added dye (Fig. 5b, c), testing by addition of heat killed bacteria (HK BACT), therefore no valid detection of staining was possible. Destaining enhancing effect of acetosyringone (and other phenolic compounds) is known to facilitate destaining of even recalcitrant dyes by aggressive oxidation. AS seems to be a superior mediator of bleaching as compared to other tested molecules [15]. Because the redox potential in the AS + H_2_O_2_ + POX mixture returns to nearly basal level after 2–3 h [16], we added the fluorescent dyes 3 h after adding the reaction mixtures to the wells. This allowed detection of SYTOX Green and DIBAC_4_(3) fluorescence in the HK BACT + H_2_O_2_ + POX and HK BACT + AS + H_2_O_2_ + POX mixtures as well (Fig. 5a, c), while in the case of TO-PRO-3 Iodide the bleaching still occurred and still no fluorescence was detectable. This phenomenon might mean that at 3 h there is still significant remaining oxidizing capacity in the H_2_O_2_ + POX and AS + H_2_O_2_ + POX mixtures that is able to bleach TO-PRO-3 (Fig. 5b), but not SYTOX Green and DIBAC_4_(3) (Fig. 5a, c). TO-PRO even seemed to go through significant fading in the control wells during the 3-h incubation. Therefore, we concluded that DIBAC_4_(3) is a suitable dye to detect bacterial cell membrane depolarization after treatment of cells by AS + H_2_O_2_ + POX mixture, given that the dye is added only 3 h after the addition of the original reaction mixture.

DIBAC_4_(3) is a voltage sensitive fluorescent dye, which can be used to monitor the dissipation of the membrane potential, not just increasing membrane permeability in general as in the case of propidium iodide (PI) or SYTOX Green dyes [30]. The latter dyes can only penetrate bacterial cells and dye the nucleoid when membrane integrity is compromised. However, the death of a cell can also result from dissipation of the transmembrane potential, which can be detected by voltage sensitive fluorescent dyes such as DIBAC_4_(3). This dye reports longer term depolarization but does not measure instant changes [31].

### Depolarization of the bacterial membrane contributes to the inhibitory effect of oxidized acetosyringone on bacteria

**Fig. 6:**
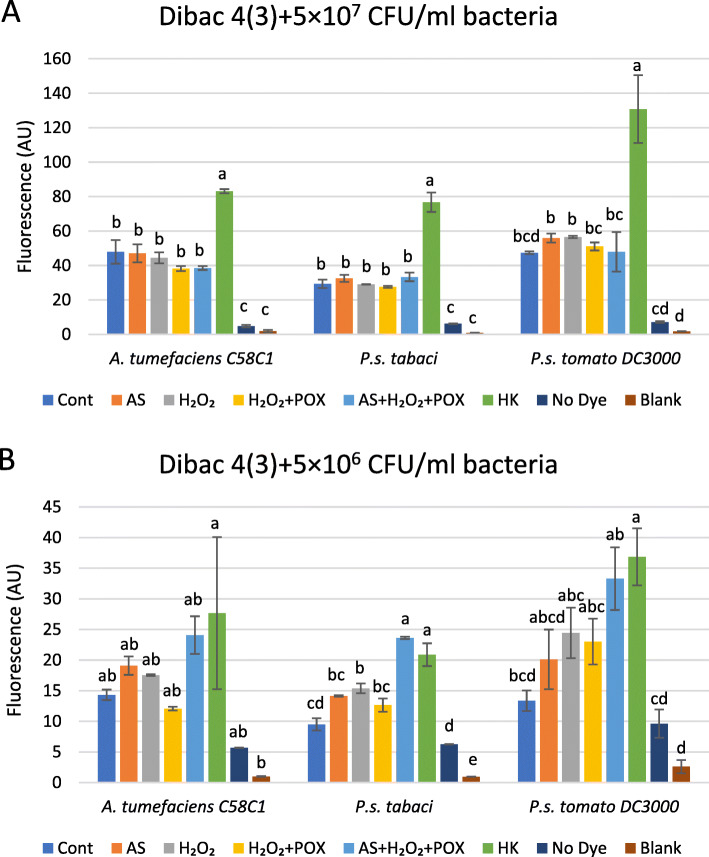
Dependence of bacterial membrane depolarisation on bacterial density after treatment with oxidized AS. Suspensions of 5 × 10^7^ (**a**) and (**b**) 5 × 10^6^ CFU/ml of *P. syringae* pv. *tabaci, P. syringae* pv. *tomato* DC3000 or *A. tumefaciens* bacteria were added to reaction mixtures containing 50 μM AS, 50 μM H_2_O_2_, and 0.72 U/ml POX, and various control mixtures from which one or two components were omitted. Bacterial membrane polarity was tested with the voltage sensitive DIBAC_4_(3) stain added 3 h after treatments of bacteria. Error bars indicate standard deviations. Different letters above the bars denote different levels of DIBAC_4_(3) fluorescence assigned by Tukey’s Test (*p* < 0.05). Abbreviations: Cont: non-treated control bacteria, AS: acetosyringone, HK: heat killed, POX: horseradish peroxidase, Blank: buffer only

We compared elevations of DIBAC_4_(3) fluorescence values in control and AS mix-treated bacterial suspensions of three species at two cell concentrations. Bacteria were incubated in the mixtures for 3 h, then DIBAC_4_(3) was added, finally, fluorescence was recorded after 1 h of co-incubation (Fig. 6a-b). The AS mixture affected fluorescence differently at the two bacterial concentrations used. At 5 × 10^7^ CFU/ml there was none or just a small elevation in fluorescence as compared to the untreated control in any of the bacteria (Fig. 6a). In contrast, at 5 × 10^6^ CFU/ml the fluorescence of the AS mix-treated bacteria was closer to the values of the heat killed controls (Fig. 6b). This correlated with the previous finding, that higher concentrations of bacteria were less affected by the same AS mix as far as the drop in colony forming units is concerned (section 2.3). Reading of fluorescence below 5 × 10^6^ CFU/ml bacterial density was not sensitive enough, therefore, we used microscopy to detect fluorescent cells in more diluted suspensions.

**Fig. 7:**
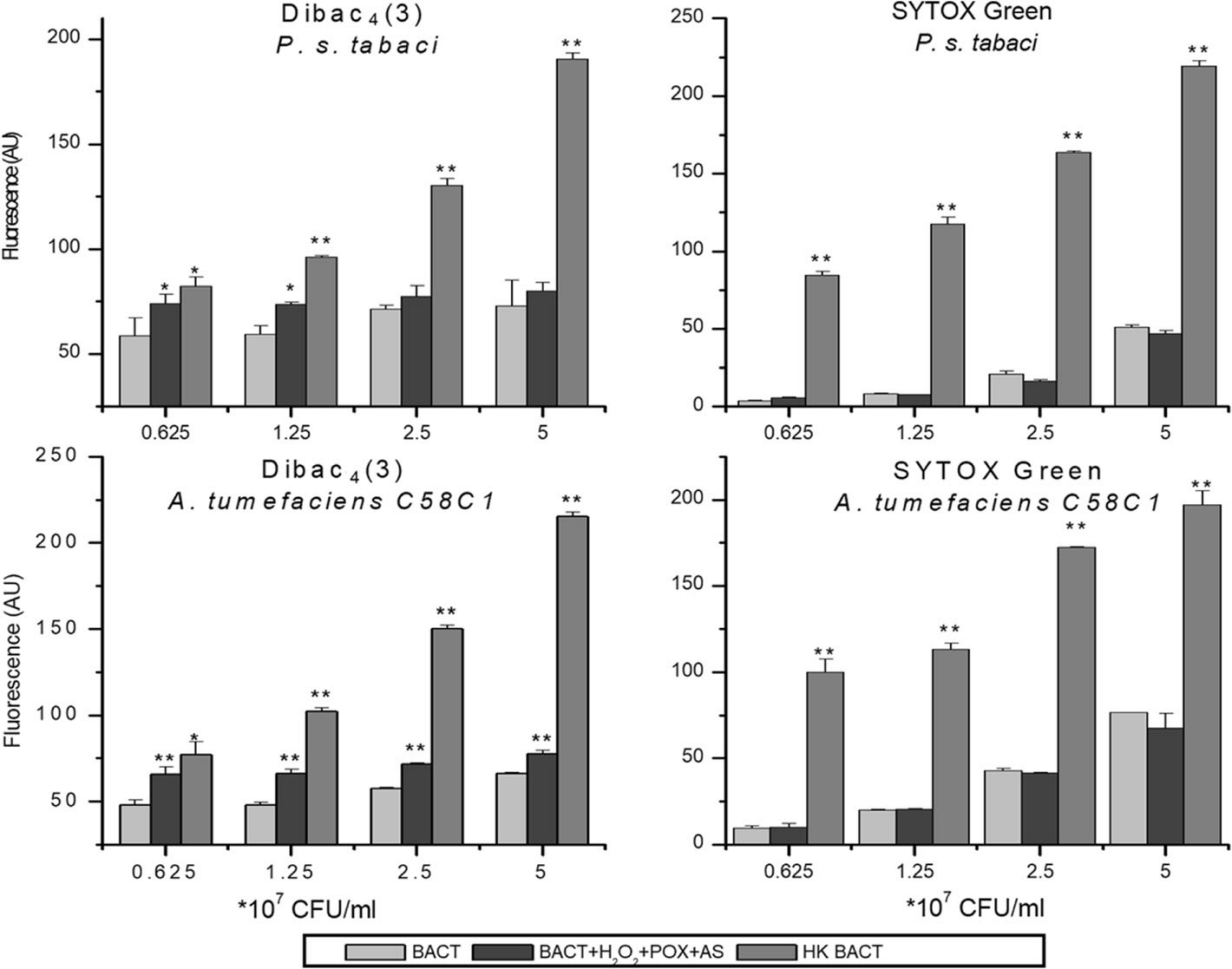
Membrane depolarization and permeability testing of bacteria after treatment with oxidized AS as a function of bacterial concentration. Dilution series of bacteria from 5 × 10^7^ to 0.625 × 10^7^ CFU/ml of *P. syringae* pv. *tabaci* and *A. tumefaciens* C58C1 bacteria was added to reaction mixtures containing 50 μM AS, 50 μM H_2_O_2_, and 0.72 U/ml POX. Untreated bacteria were used as negative, and heat killed bacteria as positive controls. Bacterial membrane polarity was tested with the voltage sensitive DIBAC_4_(3) stain, and membrane permeability with SYTOX Green stain, both added 3 h after treatment of bacteria. Error bars indicate standard deviations. Asterisks indicate significant difference from corresponding controls according to student’s T-test (*p < 0.1; **p < 0.05). Abbreviations: BACT: bacterium, AS: acetosyringone, HK: heat-killed, POX: horseradish peroxidase, AU: arbitrary unit

Corresponding literature suggests that DIBAC_4_(3) is correlated to bacterial cell death in many, but by far not all cases, as membrane depolarisation does not necessarily mean full permeabilization of the membrane [30]. Therefore, to address this issue in the case of the AS mix, we found it necessary to carry out parallel experiments with a dye that measures cell viability by detecting membrane permeability to larger molecules, such as SYTOX Green (Fig. 7).

To test, if the level of cell membrane depolarisation correlates to or diverges from cell membrane permeability when AS mix is added to plant pathogenic bacteria, the same AS mix was applied to bacterial suspensions with increasing densities. A *Pseudomonas* and an *Agrobacterium* strain were randomly selected as representative strains. Heat killed bacteria were used as positive controls both for membrane depolarisation (DIBAC_4_(3)) and membrane permeability (SYTOX Green). At lower bacterial densities (0.625 × 10^7^ and 1.25 × 10^7^ CFU/ml), significant difference was detected between DIBAC_4_(3) fluorescence of non-treated and AS mix-treated bacteria. This difference diminished towards higher bacterial densities, in the case of both bacterial species, and became insignificant in the case of *Pseudomonas syringae* pv. *tabaci*. These data indicated that membrane depolarisation is counteracted by bacteria, using an unknown mechanism. In contrast to DIBAC_4_(3), SYTOX Green fluorescence did not increase upon treatment with the AS mix. This was the case with both bacteria at all densities. Therefore we concluded that the AS mix might induce depolarization of the bacterial membrane, when bacterial density is relatively low, compared to the concentration of the components of the acetosyringone mix. On the other hand, no permeabilization of the bacterial membrane occurs.

### Microscopic evaluation of cell viability

Fluorescent microscopy was used to augment fluorescence measurements to visualize the depolarization of the bacterial cell membrane at low CFU and to compare this to the degree of membrane permeabilization, if detectable. The latter has been investigated by Postnikova et al. [32] on *P. syringae* pv. *syringae* using LIVE/DEAD® BacLight fluorescent stain, which utilizes propidium iodide (PI) to detect membrane permeability. The authors there found no permeabilization of the bacterial membrane by oxidized AS.

**Fig. 8:**
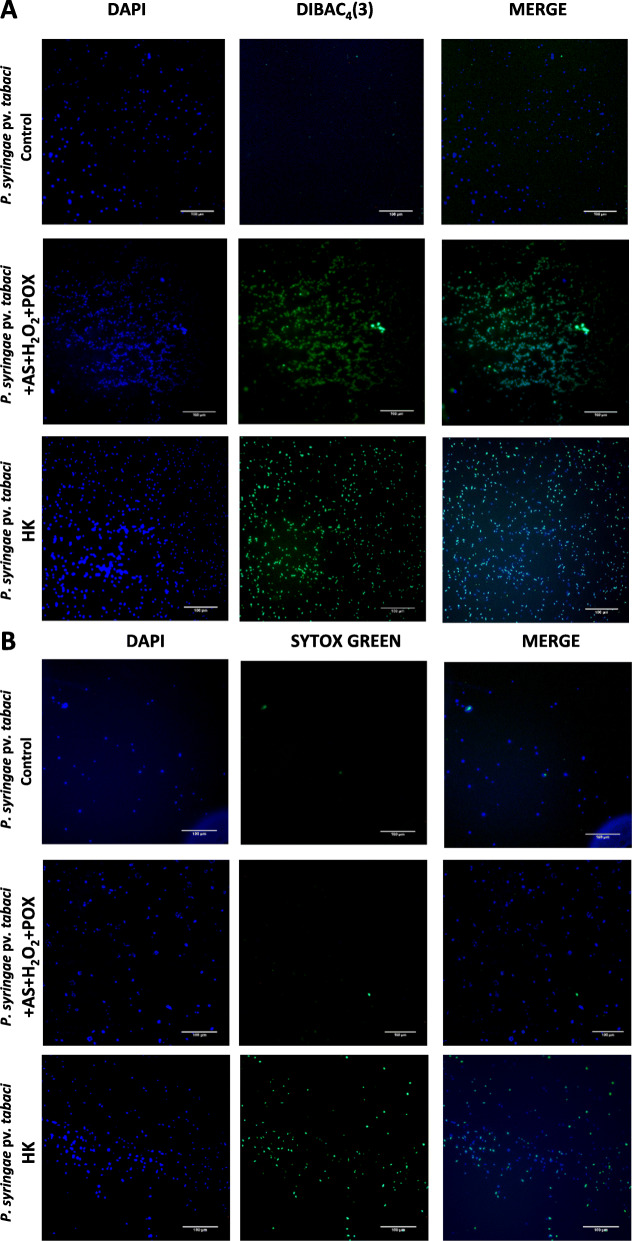
Fluorescent microscopic images of *P. syringae* pv. *tabaci* bacteria treated with an AS mix, and stained to evaluate changes in membrane permeability and polarity. *P. syringae* pv. *tabaci* (10^5^ CFU/ml) was treated with a mixture of AS + H_2_O_2_ + POX. After 3 h of shaking, (**a**) DIBAC_4_(3), an indicator dye of membrane depolarization or (**b**) SYTOX Green, an indicator of membrane permeability, and DAPI as counterstain were added and co-incubated for 1 h. After concentration of the bacteria, microscopic slides were prepared and photographed within 1–2 h. Bar = 100 μm. Abbreviations: Control: untreated bacteria AS: acetosyringone, HK: heat-killed, POX: horseradish peroxidase

Here we used the same dyes as in section 2.5., as both dyes have been shown before to be suitable for microscopy of bacteria. The voltage sensitive dye Dibac_4_(3) is known not to inhibit bacterial growth and proliferation, therefore it has even been used for time lapse microscopy [30]. SYTOX Green has widely been applied for viability staining of bacteria. We used each dye with DAPI as a counterstain to envisage all bacteria in the microscope’s field of view. As shown earlier, the inhibitory effect of the AS mix is inversely proportional to bacterial counts, therefore we worked here with a low (10^5^ CFU/ml) density of *P. syringae* pv. *tabaci* bacteria, similarly to the CFU reduction experiments (Additional file 2). Cells were incubated in the AS mix or buffer as control for 3 h, and dyes were added only then to prevent bleaching by oxidation (Fig. 8). DIBAC_4_(3) fluorescence was absent in untreated control samples, however it was remarkably strong in the AS mix-treated samples and in the heat-killed samples (Fig. 8a). This means that bacterial membranes are depolarized when the cells are incubated in the AS mix. In contrast, SYTOX Green, the dye specific for permeabilized membranes left the AS mix-incubated bacteria unstained, meaning that the membranes did not become permeable to larger molecules (Fig. 8b). The results of microscopy experiments supported those obtained by fluorescence measurements. Taken together, AS in combination with hydrogen peroxide and horseradish peroxidase resulted in membrane depolarization, but negligible membrane permeability, based on staining with specific fluorescent dyes.

### Investigation of the *in planta* effect of AS mix on compatible *P. syringae* pv. *tabaci*

**Fig. 9:**
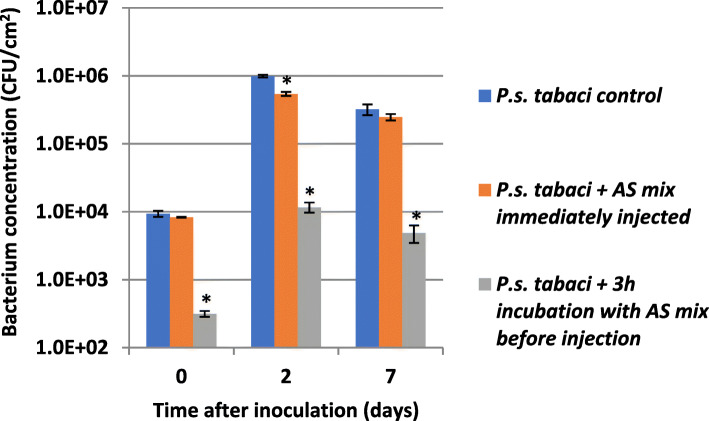
Proliferation of compatible *P. syringae* pv. *tabaci* (10^6^ CFU/ml inoculum) bacteria in tobacco (*Nicotiana tabacum*). Bacteria were suspended in 0.01 M potassium phosphate buffer pH 6. Mixture of AS+H_2_O_2_ + POX was added to the bacteria. *N. tabacum* leaves were infiltrated either immediately or after 3 h of incubation. Samples for bacterial counts were taken at 0, 2, 7 dpi. Error bars indicate standard deviations. Significant difference from untreated bacterial control according to student’s T-test is marked by a * (*p* < 0.1). Abbreviations: AS mix: acetosyringone combined with hydrogen peroxide and horseradish peroxidase, HK: heat killed, POX: horseradish peroxidase

We carried out in vivo experiments to see if external administration of the AS mix enhances the ability of tobacco plants to withstand the attack of compatible *P. syringae* pv. *tabaci*. Bacteria were suspended in 0.01 M potassium phosphate buffer (pH 6) to 10^6^ CFU/ml. AS mix components (AS, hydrogen peroxide, horseradish peroxidase) were added to final concentrations used in vitro (in section 2.4.). Then the prepared bacterial mixtures were injected into interveinal areas of tobacco leaves either immediately or after 3 h of incubation to see if the reaction mixture is able to inhibit bacterial cells in the intracellular space. Bacteria were re-isolated from the leaf tissue by grinding the leaves in a mortar and colony forming units were counted by dilution plating (Fig. 9).

Our results indicated that while the applied AS mix effectively lowered colony forming unit numbers of *P. syringae* pv. *tabaci* bacteria by cca. 2 orders of magnitude during the 3 h of incubation, no reduction in colony number was observed when the mixture was immediately injected into the leaf. *P. syringae* pv. *tabaci* bacteria proliferated at similar pace irrespective of the type of pre-treatment. In each case, bacterial counts increased about 1.5–2 orders of magnitude in the first two days, then declined slightly by the seventh day. The in vitro pre-treated bacteria could not reach a relatively higher apparent rate of proliferation as compared to the non-treated and the immediately injected bacteria. The ratio of colony forming units re-isolated from the plants inoculated with the non-treated control over that of the 3 h-preincubated bacteria increased from 30 to 65-fold by the end of the 7th day. This tells us, that if part of the bacteria were in VBNC state, they were apparently not able to return to normal state within the plant tissue, under the applied circumstances.

## Discussion

Our results indicate that AS, a tobacco metabolite specifically appearing in *N. tabacum* and *N. benthamiana* after PTI induction either by HR-negative mutant *P. syringae* pv. *syringae hrcC-* bacteria or the flg22 elicitor peptide at the time point (5–6 hpi) when PTI starts to be effective against bacterial multiplication, causes a rapid inactivation of certain plant pathogenic bacteria in vitro, when in an oxidative environment.

During our present study, AS was only detected in the PTI-induced (flg22 and *P. syringae hrcC*-) samples at 6 hpi. This was especially interesting, as it was found to rapidly inhibit several phytopathogenic *Pseudomonas* bacterium species, when combined with hydrogen peroxide and horseradish peroxidase in vitro. These results are even more interesting as over-expression of C4H (cinnamate 4-hydroxylase) has been shown to increase the accumulation of AS in elicited tobacco cell-suspension cultures [33]. Moreover, an OMT (*O*-methyltransferase) capable to synthesise AS was found in methyl jasmonate-treated tobacco cell-suspension cultures [34]. These changes in acetosyringone levels would be worth to be analyzed in the future in C4H and OMT overexpressing plants.

We demonstrated that the antibacterial effect of the AS mix is inversely proportional to bacterial density. Moreover, some *Agrobacterium* isolates are more resistant to the AS mix than the remaining plant pathogenic bacteria tested. A straightforward explanation to these phenomena could be the antioxidant capacities possessed by bacteria, e.g. the constitutive and inducible catalase enzyme activities. Xu and Pan [35] have shown that an *Agrobacterium* strain deficient in catalase activity was highly attenuated in the ability to cause tumours on plants compared with the wild type. Thus, catalase was considered a virulence factor of *Agrobacterium*, and our results might provide a possible confirmation for an antioxidant enzyme being a virulence factor, however further investigations are needed. *Agrobacteria* are known to have a peroxide and phosphate limitation-inducible catalase gene (KatA), which was also shown to be regulated by a negative feedback loop [36, 37]. Complex regulatory mechanisms may eventually lead to higher resistance of *Agrobacteria* to oxidative stress. *Pseudomonas* species and other plant pathogens are also known to have functioning catalase and OxyR (a hydrogen peroxide-sensing transcriptional activator) [38], moreover, *Pseudomonas syringae* catalases were shown to be necessary for plant pathogenesis [39].

We investigated two possible antibacterial mechanisms of the AS mix using fluorescent dyes as reporters of membrane permeabilization and membrane depolarization. We found that AS in combination with hydrogen peroxide and horseradish peroxidase resulted in membrane depolarization, but negligible membrane permeability. Our results were in concordance with those of Baker and colleagues [14] who have shown in flow cytometry experiments that PI was not able to penetrate *Pseudomonas syringae* pv. *syringae* bacterial cells after treatment with the AS mix. They also have shown that these bacterial cells reached a viable but non-culturable (VBNC) state, supported by sustained cellular respiration. Our present data imply that this perceived VBNC state is reached following significant depolarization of the bacterial cell membrane. Mariano et al. [40] made similar observations, when investigating a toxin (Ssp6) secreted by *Serratia marcescens*, that was able to cause depolarization of target cells as detected by DIBAC_4_(3), without increasing membrane permeability for larger dye molecules such as PI, similarly to SYTOX Green in our present study. In another study, Spindler et al. [41] reported an antimicrobial mechanism where membrane depolarization and permeabilization were not correlated. The peptide antibiotic Bac8c caused almost immediate but reversible membrane depolarization of the Gram-negative bacterium *Escherichia coli* at the IC50 concentration while no increase in membrane permeability was detected. At a higher concentration, depolarisation of the bacterial membrane still occurred within 5 min, and in that case, membrane permeabilization also followed, but only within 30 min. Our results underline that membrane depolarization and membrane permeabilization might be present simultaneously in the activity mechanism of antimicrobial agents or these two effects might be decoupled as in the present case of oxidized AS.

Non-impaired proliferation of the AS mix treated, and immediately injected compatible bacteria *in planta* indicates that there might be one or more factors in the plant intercellular space that either scavenges hydrogen peroxide fast, and/or metabolizes or binds free AS. The provided 50 μM concentration of H_2_O_2_ in the AS mix might prove insufficient in vivo. In resistant interactions, plants might produce significantly higher amounts at the location of bacterial invasion. ROS production has especially been widely investigated during defence reactions (Reviewed in [42]). Respiratory burst associated with pathogen attack is thought to have a signalling role, moreover, ROS might also contribute to direct antimicrobial effects in different pathosystems [43, 44]. Oxidative burst in *Arabidopsis* produced by apoplastic peroxidases has also been shown as an essential part of resistance [45]. Reactive oxygen species (ROS) producing cell wall peroxidases and a plasma membrane-localized NADPH oxidase, which are active during fungal infection, were recently identified by Kámán-Tóth and colleagues [46] in *Arabidopsis*.

Our group reported induction of peroxidase genes and peroxidase activity during PTI in tobacco leaves earlier [3, 8]; as well as localised production of hydrogen peroxide in the cell wall adjacent to the attachment site of PTI-inducing *hrpL* mutant of *Pseudomonas phaseolicola* in *N. tabacum* using electron microscopy [22]. Co-localized accumulation of H_2_O_2_ and peroxidases at the site of bacterial attachment to the plant cell wall and around bacteria themselves were also reported in leaves of various plants after infection with different bacteria including *hrp* mutant pathogens [47, 48]. These microscopic phenomena with the simultaneous accumulation of AS suggest that the conditions might be given locally for an antibacterial mix that, as we have shown, can be effective against wide range of bacterial pathogens. The in planta role of AS is however not clear at the moment, therefore further investigations would be necessary. Based on the above cited literature one possibility is the formation of local points of high antimicrobial activity near the cell wall peroxidases or NADPH oxidases, when AS concentration is also elevated in the intercellular space is most probable.

## Conclusion

During this work, we managed to reveal an indicator metabolite of pattern triggered immunity of *Nicotiana* plants. Modeling the oxidizing milieau of the intercellular space of plants invaded by PTI-inducing bacteria in vitro, we demonstrated the ability of acetosyringone combined with hydrogen peroxide and peroxidase to inhibit the growth of several different plant pathogenic bacteria. We have also shown that metabolic inhibition of the bacteria is almost immediate, moreover, that the antimicrobial effect is not based on membrane permeabilization, but on membrane depolarization of bacteria.

Based on our results we propose that besides some phenolic acids directly inhibiting bacterial growth, it seems that the interplay of a phenolic compound (e.g. acetosyringone) and the oxidative burst (generated by hydrogen peroxide and peroxidase) might enhance antibacterial activity. Investigation of the in vivo role of AS, and its interrelation with other components of the complex ROS production system of plants however will require more complex methods in the future.

Our results add a new aspect to the types of of antimicrobial activities found in plants. Direct antimicrobial activity of metabolites derived from plants have been widely investigated (reviewed in [49]). Antimicrobial activity of ROS in plants has long been studied as well [50, 51]. Enhanced antimicrobial activity resulting from the reaction of secondary metabolites and hydrogen peroxide, leading to strong antimicrobial activity is a relatively novel area that is worth extensive investigation. Plant metabolites that are produced after specific induction, like PTI elicitors in this case, might be a rich source of compounds with indirect antimicrobial activity.

## Methods

### Plant material

Tobacco plants (*Nicotiana tabacum* cv. Samsun and *Nicotiana benthamiana*) were grown in the greenhouse in soil (General potting mix from peat, clay and cow manure (Florimo® általános virágföld) pH 6.4+/− 0.5, Matécsa Ltd., Kecel, Hungary, completed with 5 V/V% perlite (Florimo® Kertészeti Perlit, 2-6 mm, Matécsa Ltd., Kecel, Hungary). Two days before and during the experiments, the 2–2.5-month-old tobacco plants were kept in a growth chamber with 16/8 h light/dark period at 23 °C. Hypodermic syringes fitted with a 25 gauge needle were used for the infiltration of the 2–3 middle leaves with bacterial suspensions or solution of flg22 peptide. At the appropriate time points, leaf samples were frozen immediately in liquid nitrogen and stored at − 70 °C until processing. Water-infiltrated adjacent leaves next to the treated ones (in the case of *N. benthamiana*) or fully separated interveinal areas on the same leaves (in the case of *N. tabacum*) were used as control. Every experiment was carried out on at least two plants in parallel as biological replications. The experiments were also repeated at least twice using different plant generations to confirm the detected trends.

### Bacterial and chemical treatments

All bacterial strains (Table 1) were cultured at 28 °C on King’s medium B [55]. Sources and reference publication of each bacterial strain is included in Table 1. Overnight cultures of bacteria were suspended in distilled water and adjusted to OD 1.0 or OD 0.21 at 560 nm (10^9^ or 10^8^ CFU/ml). Bacteria were diluted to the required density from this stock. Bacterial density was routinely verified by the plate dilution method before carrying out the experiments. Briefly, 10 μl from the 10 times dilution of the 10^5^ CFU/ml suspension was plated before starting the experiments, and colonies were counted 48 h later. The flg22 peptide (Genescript, USA) was dissolved in sterile double-distilled water to a stock solution of 1 mM. This was further diluted in sterile double-distilled water to 50 μM to infiltrate *N. benthamiana* plants.

**Table 1:** Bacterium strains used in this study

Strain	Pathogenicity / interaction type	Notes	Source and reference
*Pseudomonas syringae* pv*. syringae* 61	HR causing in tobacco, Incompatible	Gram-negative	Prof. Dr. A. Collmer, Cornell University, Ithaca, USA [52];
*Pseudomonas syringae* pv*. syringae* 61-1530B	PTI causing in tobacco, No symptoms	Gram-negative *hrcC-* mutant strain	Prof. Dr. A. Collmer, Cornell University, Ithaca, USA [5];
*Pseudomonas syringae* pv*. tabaci* H10	Compatible pathogenic on tobacco	Gram-negative	NCAIM^1^ B.01601
*Pseudomonas syringae* pv. *tomato* DC3000	Compatible pathogenic on *Arabidopsis* and tomato	Gram-negative	Prof. Dr. A. Collmer, Cornell University, Ithaca, USA [53];
*Pseudomonas syringae* pv. *tomato* DC3000 Lux	Compatible pathogenic on *Arabidopsis* and tomato	Gram-negative, with insertion of the *luxCDABE* operon from *Photorhabdus luminescens*	Prof. Dr. Julia Vorholt, ETH Zurich, Switzerland [29, 54];
*Clavibacter michiganensis* subsp. *michiganensis*	Tomato pathogen	Gram-positive	NCAIM^1^ B.01276
*Curtobacterium flaccumfaciens* pv*. flaccumfaciens*	Bean pathogen	Gram-positive	NCAIM^1^ B.01609
*Pectobacterium atrosepticum*	Potato pathogen	Gram-negative (previously *Erwinia carotovora* pv. *atroseptica*)	NCAIM^1^ B.01611
*Pectobacterium carotovorum* subsp*. carotovorum*	Diverse host range (beet, potato etc.)	Gram-negative (previously *Erwinia carotovora*)	NCAIM^1^ B.01109^T^
*Xanthomonas campestris* pv. *campestris*	Pathogen of cabbage and other crucifers	Gram-negative	NCAIM^1^ B.01224
*Agrobacterium tumefaciens* C58C1	Disarmed laboratory strain	Gram-negative (updated name *Rhizobium radiobacter*)	Dr. Sándor Süle, Plant Protection Institute, ELKH Centre for Agricultural Research, Budapest, Hungary [25];
*Agrobacterium tumefaciens* 15	Wild type isolate from cherry	Gram-negative (updated name *Rhizobium radiobacter*)	Dr. Sándor Süle, Plant Protection Institute, ELKH Centre for Agricultural Research, Budapest, Hungary [24];
*Agrobacterium tumefaciens* 3	Wild type isolate from sour cherry	Gram-negative (updated name *Rhizobium radiobacter*)	
*Agrobacterium tumefaciens* B6	Wild type isolate from dahlia	Gram-negative (updated name *Rhizobium radiobacter*)	

^1^National Collection of Agricultural and Industrial Microorganisms, Budapest, Hungary

### High-performance liquid chromatography-diode array detection-mass spectrometry (HPLC-DAD-MS)

For sample preparation, 100 mg leaf samples were ground with a mortar and pestle under liquid nitrogen. After addition of 500 μl 90% methanol they were heated to 70 °C for 15 min. The samples were then sonicated two times with an ultrasonic homogenizer (series 4710, Cole Parmer Instrument Co., IL, USA) at 55% for 45 s. Then the samples were centrifuged for 10 min at 13000 rpm. The supernatant was filtered through 0.22 μm pore size hydrophilic PTFE syringe filters (Gen-lab Ltd., Hungary), and an aliquot was used for subsequent analysis.

Standards containing 0.02 mg/ml neocholorogenic acid, cryptochlorogenic acid, chlorogenic acid, acetosyringone, salicylic acid, cinnamic acid, coumaric acid and caffeic acid (each from Sigma St Louis, MO, USA) were run parallelly. Gradient grade acetonitrile (Fisher Scientific, Pittsburg, PA, USA), formic acid (98–100%, Reanal, Budapest, Hungary) and pure water (purification equipment: Merck Millipore Direct-Q 3 UV system) were used in HPLC mobile phase.

HPLC-DAD-MS was used to identify and quantify phenolic compounds. The analysis was performed on an LC-MS-2020 system (Shimadzu, Kyoto, Japan) equipped with a binary gradient solvent pump, a vacuum degasser, a thermostated autosampler, a column oven, a photodiode detector and a single-quadrupole mass analyzer with electrospray ionization (ESI) interface. Chromatographic separations were carried out at 35 °C on a Reprospher 100 C18-DE column (150 mm × 3 mm ID, 5 μm particle size, Dr. Maisch, Ammerbuch, Germany) using 0.8 ml/min mobile phase flow rate and 10 μl injection volume. The gradient of 5% aqueous acetonitrile with 0.1% formic acid (A) and acetonitrile with 0.1% formic acid (B) was as follows: 0–10 min, 5–15% B; 10–17 min, 15–65% B; 17.1–19.5 min, 100% B and 19.51–22 min, 5% B. ESI worked under the following conditions: desolvation line (DL) temperature, 250 °C; heat block temperature, 400 °C; drying N_2_ gas flow, 15 l/min; nebulizer N_2_ gas flow, 1.5 l/min. Full mass scan spectra were recorded in the positive and negative ionization mode in the range of m/z 130–700 and selected-ion monitoring (SIM) acquisition was also performed detecting the base peak of phenolics found in the samples during pre-investigations. Data were acquired and processed using the program LabSolutions 5.42v (Shimadzu).

### In vitro susceptibility testing

Susceptibility of the phytopathogenic bacteria (Table 1) to oxidized AS was tested by serial dilutions and plating based on the method published by Mock et al. (2015). Briefly, acetosyringone (AS; Sigma St Louis, MO, USA) was dissolved in ethanol to gain a 50 mM stock solution, which was then diluted to 50 μM final concentration in 10 mM potassium phosphate buffer (PPB), pH 6. AS was used either alone, or in combination with 50 μM H_2_O_2_ (from 30% H_2_O_2_, Sigma, St Louis, MO, USA) and horseradish peroxidase (POX). This mixture of acetosyringone combined with hydrogen peroxide and horseradish peroxidase is referred to as acetosyringone mix (AS mix). Type VI horseradish peroxidase (P8375-2KU, Sigma) stock (720 U/ml) was prepared in 100 mM PPB, pH 6. Its final concentration was 0.72 U/ml in 10 mM PPB, pH 6.

Bacteria were grown as indicated in section 5.2., then were diluted to 10^5^ CFU/ml in 10 mM PPB, pH 6. The following treatment combinations were applied to the bacterial suspensions: 1. non-treated control; 2. AS; 3. H_2_O_2_; 4. H_2_O_2_ + POX; 5. AS + H_2_O_2_ + POX; 6. Heat-Killed (HK) at 70 °C for 10 min. Bacteria were incubated in the above treatment combinations in a thermoshaker (Stat Fax® 2200, Awareness Technology, Florida, USA) for 180 min at 27 + − 0.1 °C with continuous shaking (200 rpm), then were diluted 10 times. 10 μl of original and diluted mixtures were spread onto King’s B agar plates, and colonies were counted 2 days later. Treatments on each bacterial strain were carried out in duplicates at a time and were repeated at least twice on different days.

### Monitoring changes in the acetosyringone reaction mixture by mass-spectrometry

Testing solutions were prepared as described in section 5.4 but in 0.01 M ammonium acetate buffer pH 6. AS in combination with H_2_O_2_ or POX and AS mix were analysed by flow injection analysis using an LC-MS 2020 system (without column). Water (purified with Merck Millipore Direct-Q 3 UV system) with 2% methanol (gradient grade, Molar Chemicals, Budapest, Hungary) was used as mobile phase with 0.3 mL/min flow rate and 10 μL was injected immediately and at 5, 30, 60, 120 and 180 min after the preparation of the mixtures. The mixtures were kept at 25 °C during this time. The ESI-MS settings was the same as given in section 5.3, but full mass scan spectra were recorded in the positive and negative ionization mode in the range of m/z 150–800.

### Membrane depolarization and permeability assays

For membrane depolarization and permeability assays, bacterial density was adjusted to given values between 5 × 10^6^ and 5 × 10^7^ CFU/ml. Testing solutions and mixtures were prepared as described in section 5.4. Volumes of treatment combinations were 200 μl, experiments were carried out in 96 well microtiter plates in duplicates. Mixtures were incubated in a thermoshaker (Stat Fax® 2200, Awareness Technology, Florida, USA) for 180 min at 27 + − 0.1 °C with continuous shaking at 200 rpm. Fluorescent indicator dyes were then added, and co-incubated for 60 min.

The fluorescent dye DIBAC_4_(3) (Biotium Inc., CA, USA) stock solution was 1 mg/ml dissolved in DMSO. This was further diluted 1:1000 in the experimental mixture.

SYTOX® Green and TO-PRO®-3 fluorescent dyes were from the SelectFX Nuclear Labeling Kit (Invitrogen, CA, USA). SYTOX® Green (60 μg/ml in DMSO) and TO-PRO®-3 (210 μg/ml in DMSO) stock solutions were both diluted 1:300 as recommended by the manufacturer.

Fluorescence was then recorded by an iBright™ FL1000 Imaging System (Thermo Fisher Scientific Inc. MA, USA) in the GFP channel. Quantification was performed using the NIH ImageJ [56] software ReadPlate2.1 plugin.

### Fluorescent microscopy

For fluorescent microscopy, bacterial density was adjusted to 10^5^ CFU/ml. Testing solutions and mixtures were prepared as described in section 5.4. Volumes of treatment combinations were 1 ml. Mixtures were incubated in a thermoshaker (Stat Fax® 2200, Awareness Technology, Florida, USA) for 180 min at 27 + − 0.1 °C with continuous shaking. Fluorescent indicator dyes were then added, and co-incubated for 60 min. Bacterial cells were then centrifuged at 8000 rpm for 1 min. Then 990 μl of the supernatant was discarded, the bacterial cells were re-suspended in the remaining 10 μl, and taken for fluorescent microscopy immediately.

The fluorescent dyes DIBAC_4_(3) and SYTOX Green were used at the same concentrations as described in section 5.6. DAPI originated from the SelectFX Nuclear Labeling Kit (60 μg/ml in DMSO; Invitrogen, CA, USA), and was used at 1:300 dilution, as recommended by the manufacturer.

Fluorescent microscopic images were collected using a Zeiss Axioskop 2 Plus fluorescent microscope (Carl Zeiss Microscopy GmbH). SYTOX Green and DIBAC_4_(3) (green fluorescence) were detected in epifluorescent mode using a filter set composed of a 450–490 nm excitation filter, a 495 nm dichroic mirror, and a 500–550 nm barrier filter. DAPI (blue) fluorescence was detected using a fluorescent filter set composed of a 365 nm excitation filter, a 395 nm dichroic mirror, and a 420–470 nm barrier filter. Overlay images were generated using NIH ImageJ software [56]. Brightness and contrast adjustments were applied across entire images and controls using NIH ImageJ. Brightness and contrast were maintained so that every captured feature remains clearly visible in each image.

### In vivo (*in planta*) bacterial proliferation testing

Tobacco plants were grown as described in 2.1. Density of *Pseudomonas syringae* pv. *tabaci* bacteria was adjusted to 10^6^ CFU/ml. Testing solutions and mixtures were prepared in 0.01 M PPB pH 6 as described in section 5.4. Treatments were the following: **1.** bacteria (control); **2.** bacteria + 50 μm AS + 50 μm H_2_O_2_ + 0.72 U/ml POX; mixture injected immediately; **3.** bacteria + 50 μm AS + 50 μm H_2_O_2_ + 0.72 U/ml POX; mixture injected following 3 h of shaking at 27 °C. Hypodermic syringes fitted with a 25 gauge needle were used for the infiltration of the 3 middle leaves of tobacco with bacterial suspensions as described by [57]. Eight pieces of 11 mm diameter discs from three leaves per time point were homogenised in 800 μl 10 mM MgCl_2_ in a mortar. The number of viable cells was calculated by the plate-count technique on King’s B agar plates. The experiment was carried out twice with similar results.

## Supplementary Information

**Additional file 1. **Relative accumulation of AS in *N. tabacum* leaves. Response to treatments with *Pseudomonas syringae* pv. *syringae hrcC*- bacteria at 2, 4 and 6 hpi. Values are averages of three biological replicates. All values were normalized with non-treated control levels. Error bars indicate standard deviations. Asterisks indicate significant difference from corresponding water treated controls according to student’s T-test (*p < 0.1; **p < 0.05). W: water-treated control; P.s. syringae *hrcC* (HR-): *P. syringae* pv. *syringae hrcC-* (HR negative mutant).

**Additional file 2.** Antimicrobial effect of oxidized form of AS on different plant pathogenic bacteria. 5 × 10^6^ or 5 × 10^7^ CFU/ml bacteria were added to reaction mixtures containing 50 μM acetosyringone, 50 μM H_2_O_2_, and 0.72 U/ml horseradish peroxidase, and control mixtures from which acetosyringone was omitted. Serial dilutions were plated following 3 h of co-incubation, for CFU determination. A-D) Diagrams showing quantification of the results. Error bars indicate standard deviations. Abbreviations: Bact: bacterium, AS: acetosyringone, HK: heat-killed, POX: horseradish peroxidase.

**Additional file 3.** LC–mass spectrometry analysis of the AS MIX reaction in a time course experiment. Relative quantity of AS and new compounds in the reaction mixtures containing 50 μM acetosyringone, 50 μM H_2_O_2_, and 0.72 U/ml horseradish peroxidase, and control mixtures from which H_2_O_2_ or horseradish peroxidase was omitted. Reaction mixtures were tracked through 180 min using HPLC-MS. Compounds are denoted by to their m/z values. Abbreviations: AS: acetosyringone, POX: horseradish peroxidase

## Data Availability

The datasets used and/or analysed during the current study are available from the corresponding author on reasonable request.

## Abbreviations

PTI: pattern-triggered immunity
AS: acetosyringone
AS mix: mixture of acetosyringone, hydrogen peroxide and horseradish peroxidase
POX: horseradish peroxidase
PRRs: plant resistance recruits pattern recognition receptors
TLRs: Toll-like receptors
MAMPs: microbe associated molecular patterns
EF-Tu: elongation factor Tu
PGN: peptidoglycan
LPSs: lipopolysaccharides
MAPK: mitogen activated protein kinase
ROS: reactive oxygen species
ESTs: expressed sequence tags
HPLC-DAD-MS: high-performance liquid chromatography-diode array detection-mass spectrometry
PPB: potassium phosphate buffer
HK: heat-killed
HK BACT: heat killed bacteria
P. s. syringae: *Pseudomonas syringae* pv. *syringae*
P. s. tabaci: *Pseudomonas syringae* pv. *tabaci*
P. s. tomato: *Pseudomonas syringae* pv. *tomato*
X. c. campestris: *Xanthomonas campestris* pv. *campestris*
P. c. carotovorum: *Pectobacterium carotovorum* subsp. *carotovorum*
C. m. michiganensis: *Clavibacter michiganensis* subsp. *michiganensis*
HR: hypersensitive response
hpi: hours post inoculation
ESI: electrospray ionization
PI: propidium iodide
VBNC: viable but non-culturable
