# Supplementary material for: A pattern-triggered immunity-related phenolic, acetosyringone, boosts rapid inhibition of a diverse set of plant pathogenic bacteria
Source: BMC Plant Biol. 2021 Mar 25;21:153. doi: 10.1186/s12870-021-02928-4 (PMC7992983; doi:10.1186/s12870-021-02928-4)
Supplement: Supplementary file 1 — Additional file 1. Relative accumulation of AS in N. tabacum leaves. Response to treatments with Pseudomonas syringae pv. syringae hrcC- bacteria at 2, 4 and 6 hpi. Values are averages of three biological replicates. All values were normalized with non-treated control levels. Error bars indicate standard deviations. Asterisks indicate significant difference from corresponding water treated controls according to student’s T-test (*p < 0.1; **p < 0.05). W: water-treated control; P.s. syringae hrcC (HR-): P. syringae pv. syringae hrcC- (HR negative mutant). [file 12870_2021_2928_MOESM1_ESM.pptx]

## Slide 1
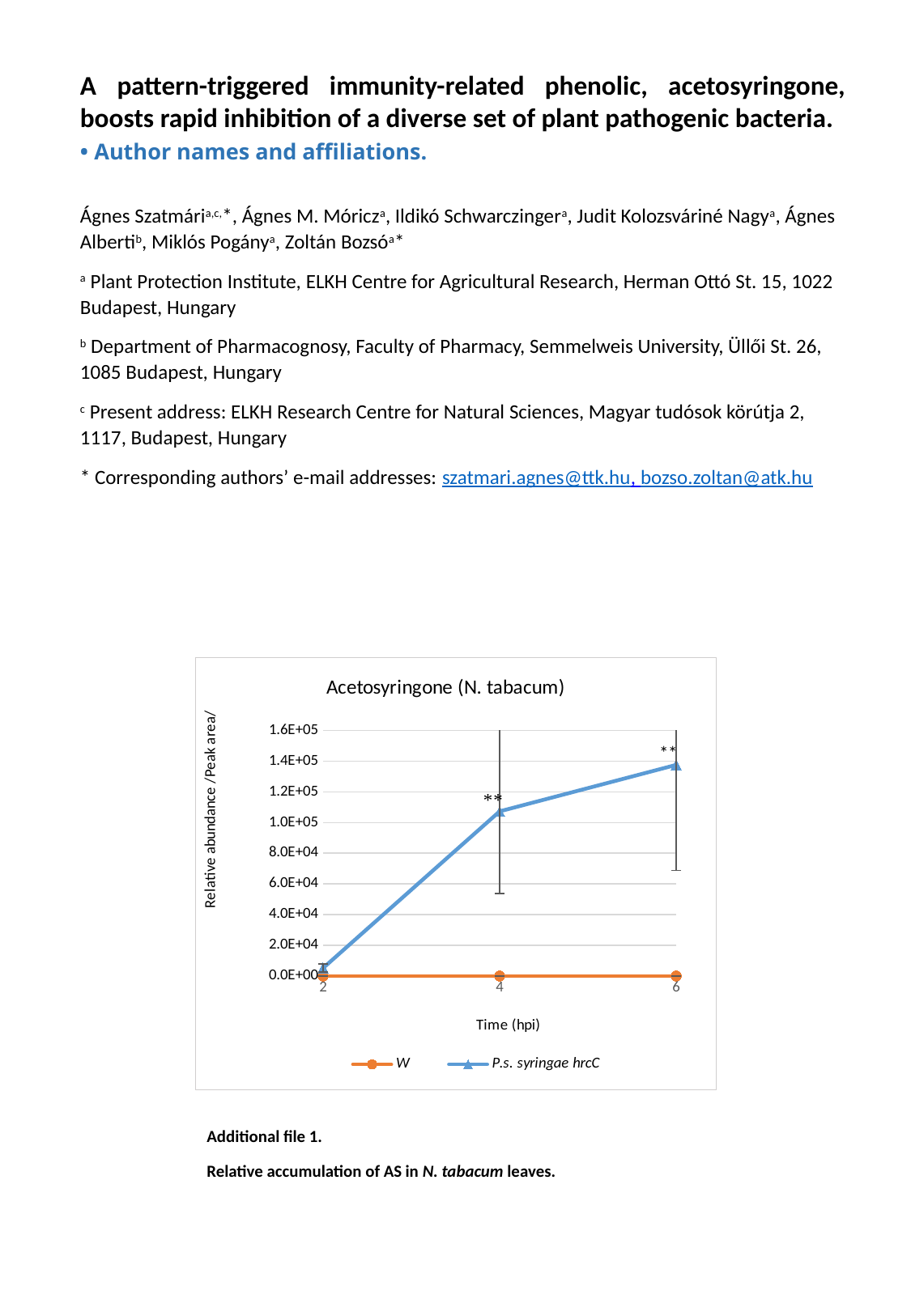

A pattern-triggered immunity-related phenolic, acetosyringone, boosts rapid inhibition of a diverse set of plant pathogenic bacteria.
• Author names and affiliations.
Ágnes Szatmária,c,*, Ágnes M. Móricza, Ildikó Schwarczingera, Judit Kolozsváriné Nagya, Ágnes Albertib, Miklós Pogánya, Zoltán Bozsóa*
a Plant Protection Institute, ELKH Centre for Agricultural Research, Herman Ottó St. 15, 1022 Budapest, Hungary
b Department of Pharmacognosy, Faculty of Pharmacy, Semmelweis University, Üllői St. 26, 1085 Budapest, Hungary
c Present address: ELKH Research Centre for Natural Sciences, Magyar tudósok körútja 2, 1117, Budapest, Hungary
* Corresponding authors’ e-mail addresses: szatmari.agnes@ttk.hu, bozso.zoltan@atk.hu
### Chart: Acetosyringone (N. tabacum)
| Category | W | P.s. syringae hrcC |
|---|---|---|
| 2 | 0.0 | 5171.333333333333 |
| 4 | 0.0 | 107281.0 |
| 6 | 0.0 | 137545.33333333334 |Additional file 1.
Relative accumulation of AS in N. tabacum leaves.
