# Supplementary material for: A pattern-triggered immunity-related phenolic, acetosyringone, boosts rapid inhibition of a diverse set of plant pathogenic bacteria
Source: BMC Plant Biol. 2021 Mar 25;21:153. doi: 10.1186/s12870-021-02928-4 (PMC7992983; doi:10.1186/s12870-021-02928-4)
Supplement: Supplementary file 2 — Additional file 2. Antimicrobial effect of oxidized form of AS on different plant pathogenic bacteria. 5 × 10^6^ or 5 × 10^7^ CFU/ml bacteria were added to reaction mixtures containing 50 μM acetosyringone, 50 μM H2O2, and 0.72 U/ml horseradish peroxidase, and control mixtures from which acetosyringone was omitted. Serial dilutions were plated following 3 h of co-incubation, for CFU determination. A-D) Diagrams showing quantification of the results. Error bars indicate standard deviations. Abbreviations: Bact: bacterium, AS: acetosyringone, HK: heat-killed, POX: horseradish peroxidase. [file 12870_2021_2928_MOESM2_ESM.pptx]

## Slide 1
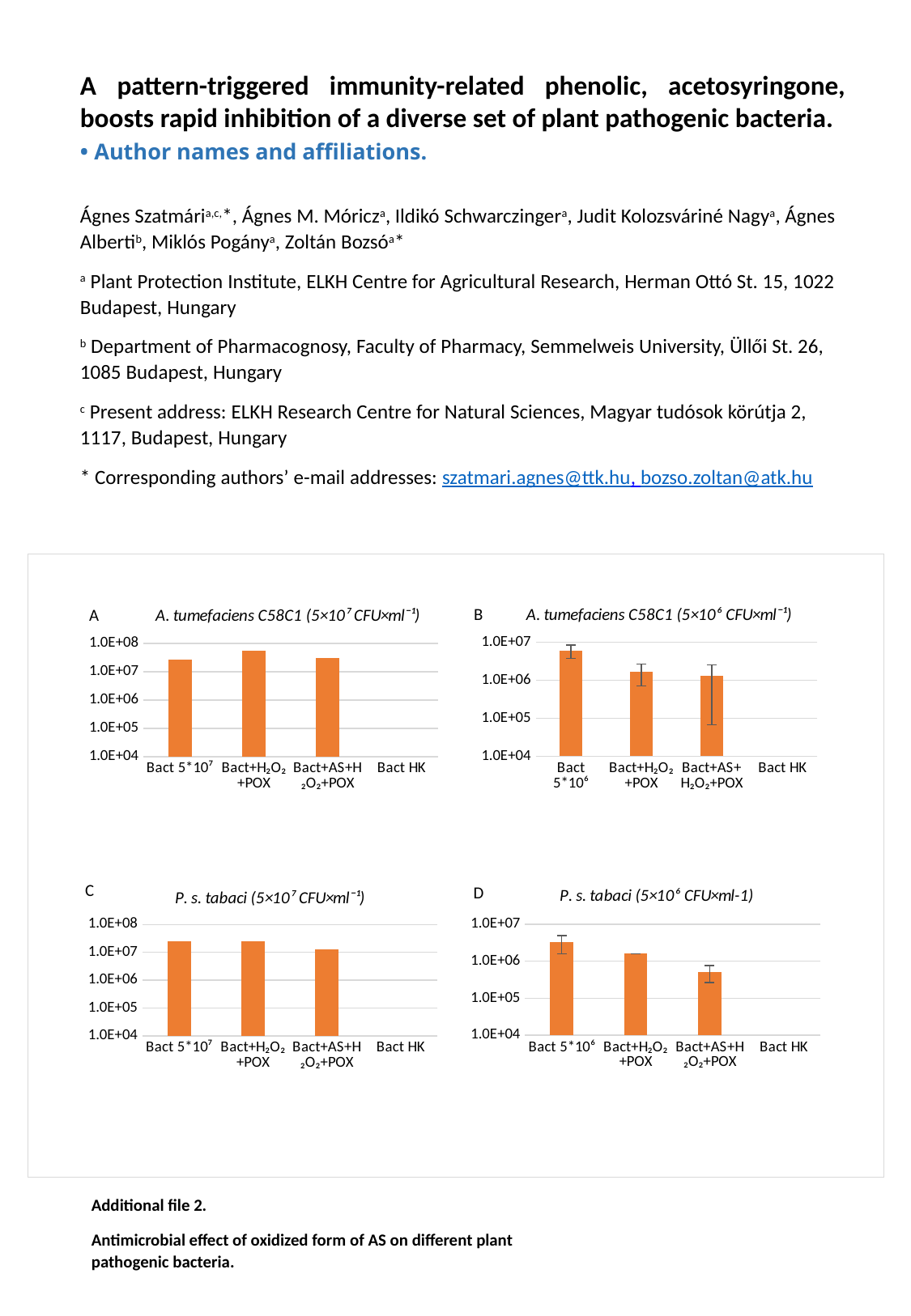

A pattern-triggered immunity-related phenolic, acetosyringone, boosts rapid inhibition of a diverse set of plant pathogenic bacteria.
• Author names and affiliations.
Ágnes Szatmária,c,*, Ágnes M. Móricza, Ildikó Schwarczingera, Judit Kolozsváriné Nagya, Ágnes Albertib, Miklós Pogánya, Zoltán Bozsóa*
a Plant Protection Institute, ELKH Centre for Agricultural Research, Herman Ottó St. 15, 1022 Budapest, Hungary
b Department of Pharmacognosy, Faculty of Pharmacy, Semmelweis University, Üllői St. 26, 1085 Budapest, Hungary
c Present address: ELKH Research Centre for Natural Sciences, Magyar tudósok körútja 2, 1117, Budapest, Hungary
* Corresponding authors’ e-mail addresses: szatmari.agnes@ttk.hu, bozso.zoltan@atk.hu
### Chart: A. tumefaciens C58C1 (5×10⁷ CFU×ml¯¹)
| Category | ÁTL |
|---|---|
| Bact 5*10⁷ | 27000000.0 |
| Bact+H₂O₂+POX | 53000000.0 |
| Bact+AS+H₂O₂+POX | 30000000.0 |
| Bact HK | 0.0 |B
A
### Chart: A. tumefaciens C58C1 (5×10⁶ CFU×ml¯¹)
| Category | ÁTL |
|---|---|
| Bact 5*10⁶ | 6125000.0 |
| Bact+H₂O₂+POX | 1700000.0 |
| Bact+AS+H₂O₂+POX | 1317500.0 |
| Bact HK | 0.0 |C
D
### Chart: P. s. tabaci (5×10⁶ CFU×ml-1)
| Category | ÁTL |
|---|---|
| Bact 5*10⁶ | 3250000.0 |
| Bact+H₂O₂+POX | 1600000.0 |
| Bact+AS+H₂O₂+POX | 515000.0 |
| Bact HK | 0.0 |
### Chart: P. s. tabaci (5×10⁷ CFU×ml¯¹)
| Category | ÁTL |
|---|---|
| Bact 5*10⁷ | 25000000.0 |
| Bact+H₂O₂+POX | 25000000.0 |
| Bact+AS+H₂O₂+POX | 13000000.0 |
| Bact HK | 0.0 |Additional file 2.
Antimicrobial effect of oxidized form of AS on different plant pathogenic bacteria.
