# Supplementary material for: A pattern-triggered immunity-related phenolic, acetosyringone, boosts rapid inhibition of a diverse set of plant pathogenic bacteria
Source: BMC Plant Biol. 2021 Mar 25;21:153. doi: 10.1186/s12870-021-02928-4 (PMC7992983; doi:10.1186/s12870-021-02928-4)
Supplement: Supplementary file 3 — Additional file 3. LC–mass spectrometry analysis of the AS MIX reaction in a time course experiment. Relative quantity of AS and new compounds in the reaction mixtures containing 50 μM acetosyringone, 50 μM H2O2, and 0.72 U/ml horseradish peroxidase, and control mixtures from which H2O2 or horseradish peroxidase was omitted. Reaction mixtures were tracked through 180 min using HPLC-MS. Compounds are denoted by to their m/z values. Abbreviations: AS: acetosyringone, POX: horseradish peroxidase [file 12870_2021_2928_MOESM3_ESM.pptx]

## Slide 1
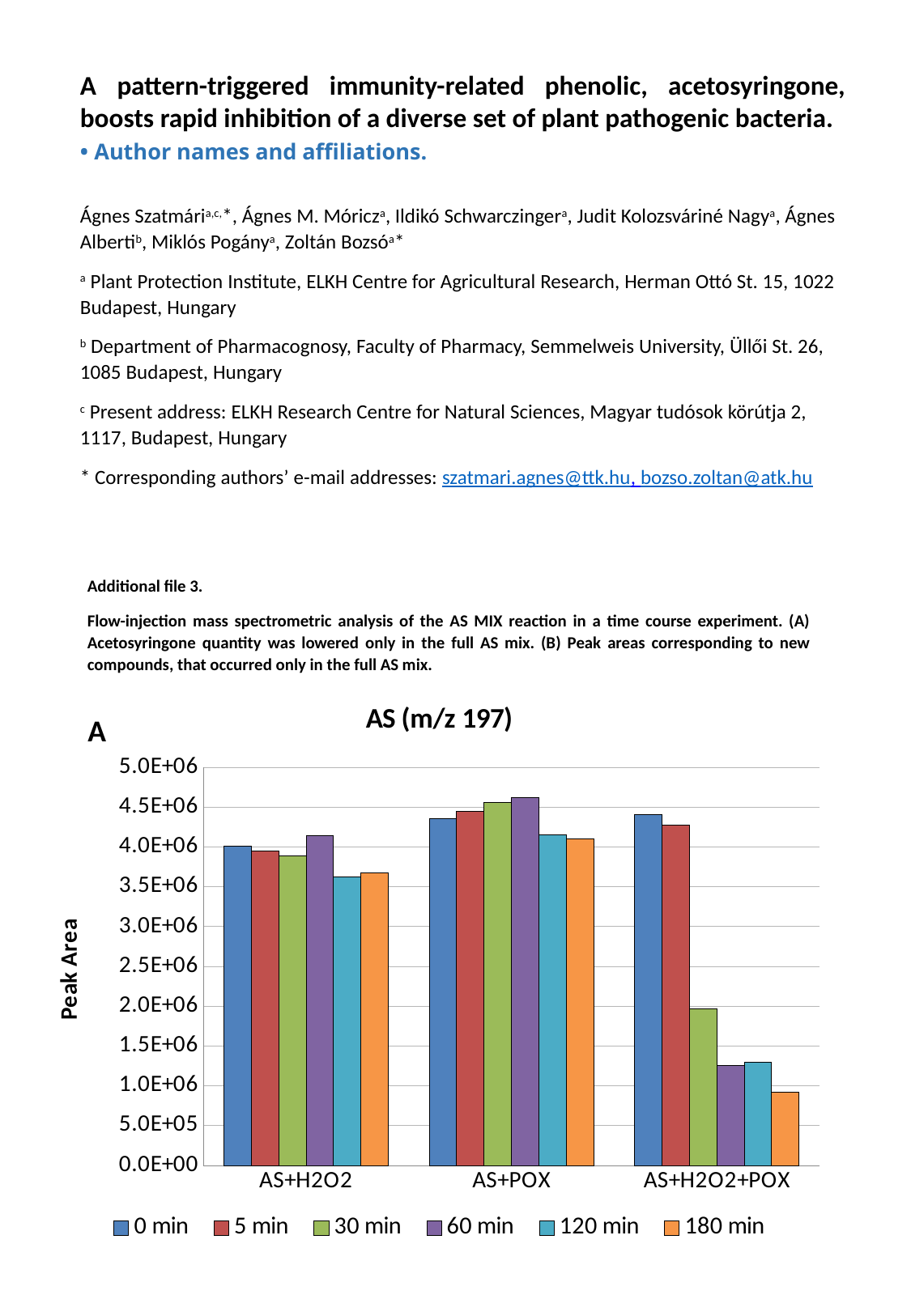

A pattern-triggered immunity-related phenolic, acetosyringone, boosts rapid inhibition of a diverse set of plant pathogenic bacteria.
• Author names and affiliations.
Ágnes Szatmária,c,*, Ágnes M. Móricza, Ildikó Schwarczingera, Judit Kolozsváriné Nagya, Ágnes Albertib, Miklós Pogánya, Zoltán Bozsóa*
a Plant Protection Institute, ELKH Centre for Agricultural Research, Herman Ottó St. 15, 1022 Budapest, Hungary
b Department of Pharmacognosy, Faculty of Pharmacy, Semmelweis University, Üllői St. 26, 1085 Budapest, Hungary
c Present address: ELKH Research Centre for Natural Sciences, Magyar tudósok körútja 2, 1117, Budapest, Hungary
* Corresponding authors’ e-mail addresses: szatmari.agnes@ttk.hu, bozso.zoltan@atk.hu
Additional file 3.
Flow-injection mass spectrometric analysis of the AS MIX reaction in a time course experiment. (A) Acetosyringone quantity was lowered only in the full AS mix. (B) Peak areas corresponding to new compounds, that occurred only in the full AS mix.
### Chart: AS (m/z 197)
| Category | | | | | | |
|---|---|---|---|---|---|---|
| AS+H2O2 | 4010334.0 | 3954538.0 | 3889001.0 | 4140166.0 | 3621870.0 | 3672862.0 |
| AS+POX | 4356406.0 | 4451216.0 | 4564842.0 | 4618718.0 | 4153763.0 | 4105922.0 |
| AS+H2O2+POX | 4403141.0 | 4272763.0 | 1968875.0 | 1255526.0 | 1297427.0 | 917349.0 |A

## Slide 2
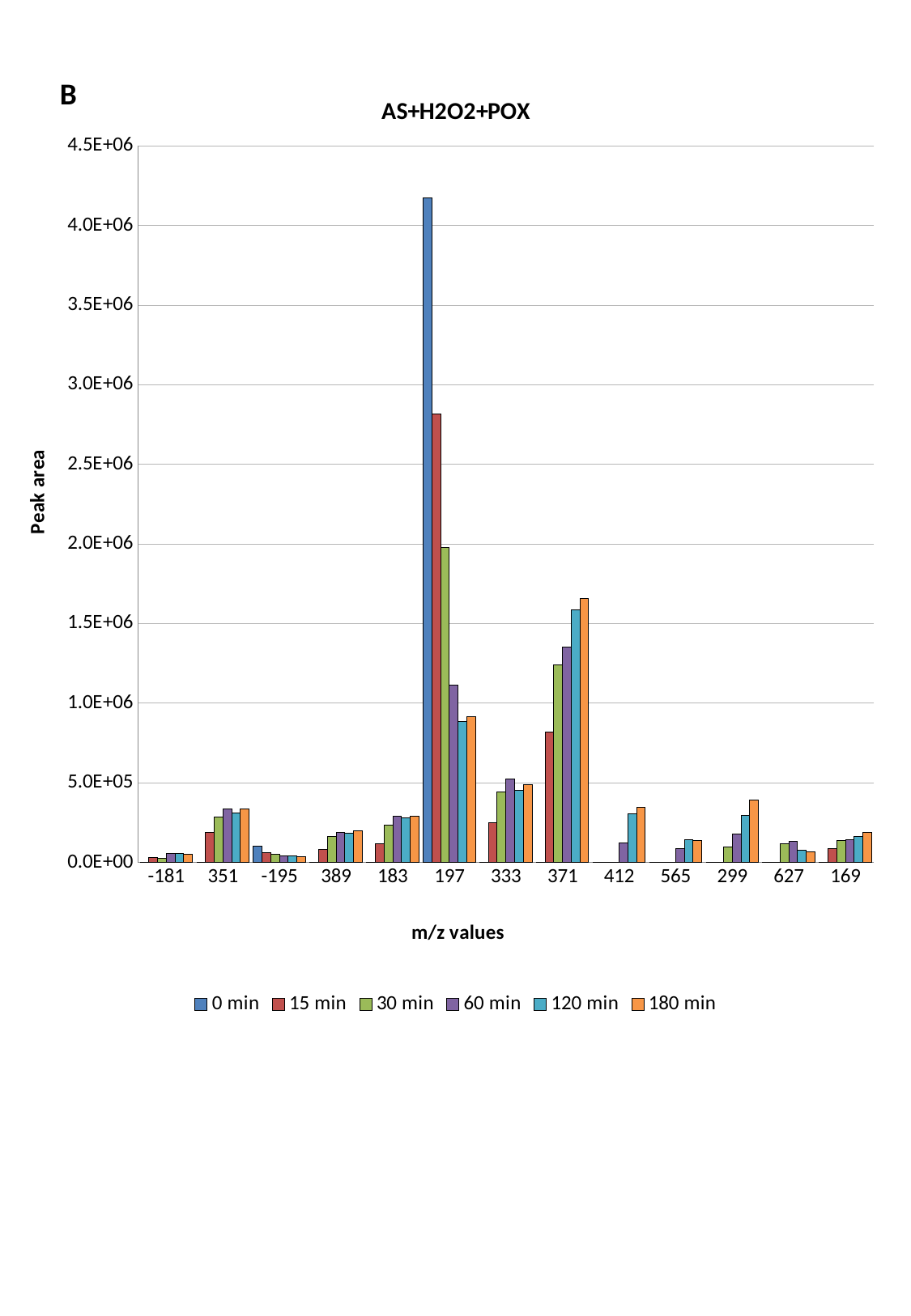

### Chart: AS+H2O2+POX
| Category | | | | | | |
|---|---|---|---|---|---|---|
| -181 | 0.0 | 29726.0 | 27220.0 | 55958.0 | 58866.0 | 54290.0 |
| 351 | 0.0 | 190240.0 | 284756.0 | 334647.0 | 312494.0 | 335039.0 |
| -195 | 104114.0 | 62822.0 | 51625.0 | 39386.0 | 42331.0 | 33968.0 |
| 389 | 0.0 | 79949.0 | 161996.0 | 188911.0 | 184210.0 | 198535.0 |
| 183 | 0.0 | 119700.0 | 233148.0 | 291962.0 | 279588.0 | 290280.0 |
| 197 | 4176554.0 | 2819539.0 | 1976811.0 | 1116728.0 | 885545.0 | 917349.0 |
| 333 | 0.0 | 247886.0 | 443351.0 | 527075.0 | 454969.0 | 490916.0 |
| 371 | 0.0 | 819132.0 | 1240320.0 | 1352794.0 | 1584925.0 | 1655695.0 |
| 412 | 0.0 | 0.0 | 0.0 | 120950.0 | 304284.0 | 346397.0 |
| 565 | 0.0 | 0.0 | 0.0 | 85459.0 | 141682.0 | 138540.0 |
| 299 | 0.0 | 0.0 | 97585.0 | 180216.0 | 293426.0 | 391747.0 |
| 627 | 0.0 | 0.0 | 116712.0 | 133454.0 | 76450.0 | 67253.0 |
| 169 | 0.0 | 89165.0 | 138961.0 | 144582.0 | 162983.0 | 189674.0 |B
